# Chimpanzee adenoviral vector prime-boost regimen elicits potent immune responses against Ebola virus in mice and rhesus macaques

**DOI:** 10.1080/22221751.2019.1644968

**Published:** 2019-07-24

**Authors:** Xi Yang, Xiang Wang, Yufeng Song, Ping Zhou, Dapeng Li, Chao Zhang, Xia Jin, Zhong Huang, Dongming Zhou

**Affiliations:** a University of Chinese Academy of Sciences, Beijing, People’s Republic of China; b Vaccine Research Center, CAS Key Laboratory of Molecular Virology and Immunology, Institut Pasteur of Shanghai, Chinese Academy of Sciences, Shanghai, People’s Republic of China; c Shanghai Institute of Rheumatology, Renji Hospital, School of Medicine, Shanghai Jiaotong University, Shanghai, People’s Republic of China; d Department of Pathogen Biology, School of Basic Medical Sciences, Tianjin Medical University, Tianjin, People’s Republic of China

**Keywords:** Chimpanzee adenoviral vector, AdC7, AdC68, prime-boost, Ebola vaccine

## Abstract

In the last few decades, Ebola virus (EBOV) has emerged periodically and infected people in Africa, resulting in an extremely high mortality rate. With no available prophylaxis or cure so far, a highly effective Ebola vaccine is urgently needed. In this study, we developed a novel chimpanzee adenovirus-based prime-boost vaccine by exploiting two recombinant replication-deficient chimpanzee adenoviral vectors, AdC7 and AdC68, which express glycoproteins (GP) of the EBOV strain identified in the 2014 outbreak. Our results indicated that a single immunization using AdC7 or AdC68 could stimulate potent EBOV-specific antibody responses, whereas the AdC7 prime-AdC68 boost regimen induced much stronger and sustained humoral and cellular immune responses in both mice and rhesus monkeys, compared with AdC7 or AdC68 single vaccination or the AdC68 prime-AdC7 boost regimen. This prime-boost vaccine could also protect mice from the simulated infection with EBOV-like particle (EBOVLP) in biosafety level 2 (BSL-2) laboratories, and antibodies from the prime-boost immunized rhesus macaques could passively provide protection against EBOVLP infection. Altogether, our results show that the AdC7 prime-AdC68 boost vaccine is a promising candidate for further development to combat EBOV infections.

## Introduction

As a single-stranded RNA filovirus, Ebola virus (EBOV) causes the severe Ebola virus disease (EVD) and subsequently led to an extremely high mortality rate in the last four decades, since its discovery in 1976 [1–3]. The recent outbreak of EBOV resulted in 28,646 EVD cases and 11,323 fatalities [4,5]. EBOV is also considered as a potential bioterrorism agent due to its high contagiousness and virulent nature [6].

Vaccination is the most cost-effective measure for the prevention and control of certain infectious diseases. A variety of EBOV vaccine candidates have been developed in past 10 years, and some of them have reached the clinical trial stage, including DNA vaccines [7], virus-like particle (VLP) based vaccines [8], recombinant vesicular stomatitis virus (rVSV) vectored vaccines [9], recombinant adenoviral vaccines [10,11], and modified vaccinia Ankara (MVA) vectored vaccines [11–13]. Among these vaccine candidates, an rVSV vector-based live attenuated Ebola vaccine developed by *Merck* & *Co*. Inc was submitted for a US Food and Drug Administration (FDA) license approval [14]. In 2017, the China FDA approved an Ebola vaccine based on an adenoviral vector originated from human adenovirus serotype 5 (Ad5) [15]. These two types of vaccines have provided basic support for the prevention and control of an Ebola epidemic in some cases. However, both vaccines involve single dose immunization, and the intensity and duration of the single dose immunization-induced immunity need to be improved. Related studies suggest that a more efficient EBOV vaccine with sustained immunity, good safety, and tolerability is needed, especially for the front-line workers [16]. Thus, the heterologous prime-boost strategy represents an innovative approach to fulfil this need [16].

Chimpanzee adenovirus rarely circulates in humans and most people do not have corresponding pre-existing immunities [17,18]. Vectors derived from chimpanzee adenovirus share desirable features with human adenovirus, such as a broad tissue tropism and ease of large-scale manufacturing [19]. Therefore, chimpanzee adenoviral vectors are widely employed in the development of various vaccines, including the EBOV vaccine [20]. A chimpanzee adenoviral vector termed as ChAd3 was considered as a replacement of Ad5 for developing an EBOV vaccine. ChAd3-based single dose EBOV vaccines or heterologous prime-boost EBOV vaccines have been tested in phase I or phase II clinical trials with promising results [21]. AdC7 and AdC68 are two chimpanzee adenovirus-modified vectors, similar to the Ad5 vector, which induces potent transgene product-specific B-cell and T-cell immune responses that are not impaired by a pre-existing immunity to common human adenovirus serotypes [18]. An AdC7-based EBOV vaccine has been tested in mice and guinea pigs previously, which induced strong immune responses and conferred complete protection. Although the results from this study were encouraging, this vaccine candidate needs to be further tested in non-human primates [22].

In this study, we aimed to assess and fully characterize the immune responses elicited by AdC7 and AdC68 as a vaccine platform against EBOV. To this end, we constructed the recombinant vectors AdC7 and AdC68 expressing the Ebola envelope GP identified in the 2014 Ebola outbreak and tested these two vaccine candidates via single dose immunization and prime-boost regimen in both murine and rhesus monkey models with simulated Ebola infection.

## Materials and methods

### Construction of recombinant adenoviral vectors AdC7 or AdC68 expressing EBOV GP

EBOV full-length GP open reading frame of Zaire strain isolated from H. sapiens-wt/GIN/2014/Makona-Gueckedou-C07 (GenBank accession number KJ660347) was codon-optimized and synthesized by GenScript (Nanjing, China). The GP gene was then inserted into pShuttle vector (Clontech Laboratories Inc., USA) by the restriction sites XbalI and KpnI. Subsequently, the whole GP expression cassette containing CMV promoter, GP gene, and BGH polyA tail was cloned into the E1- deleted region of adenoviral vectors pAdC7 or pAdC68 by I-CeuI and PI-SceI sites separately (Supplementary Figure 1A). The E1 and E3-deleted replication-deficient adenoviral vectors were generated by our lab [23,24]. AdC7-empty and AdC68-empty with no insertion in E1 deleted region were employed as two control viruses and generated as mentioned above. Recombinant adenoviruses were rescued, expanded, and purified by CsCl gradient ultracentrifugation as previously described [24]. Viral particle (vp) numbers were determined by spectrophotometry.

### *In vitro* EBOV GP expression

HEK293 cells were infected with different doses of AdC7-EBOV, AdC68-EBOVgp, AdC7-empty, and AdC68-empty. Twenty-four hours post-infection, HEK293 cells were collected, and the cell lysates underwent SDS-PAGE, followed by Western blotting with anti-EBOV GP monoclonal antibody 6D8 generated as previously described or anti-β-actin monoclonal antibody (A5441, Sigma-Aldrich, St. Louis, MO, USA) (Supplementary Figure 1B) [19].

### Animal immunization

All animal experimental protocols involved in this study were approved and followed the guidelines provided by the Institutional Animal Care and Use Committee of the Institut Pasteur of Shanghai and Non-Human Primates Experiment Platform of Institute of Neuroscience, Chinese Academy of Sciences (protocol approval number: A20160927).

**Figure 1.**
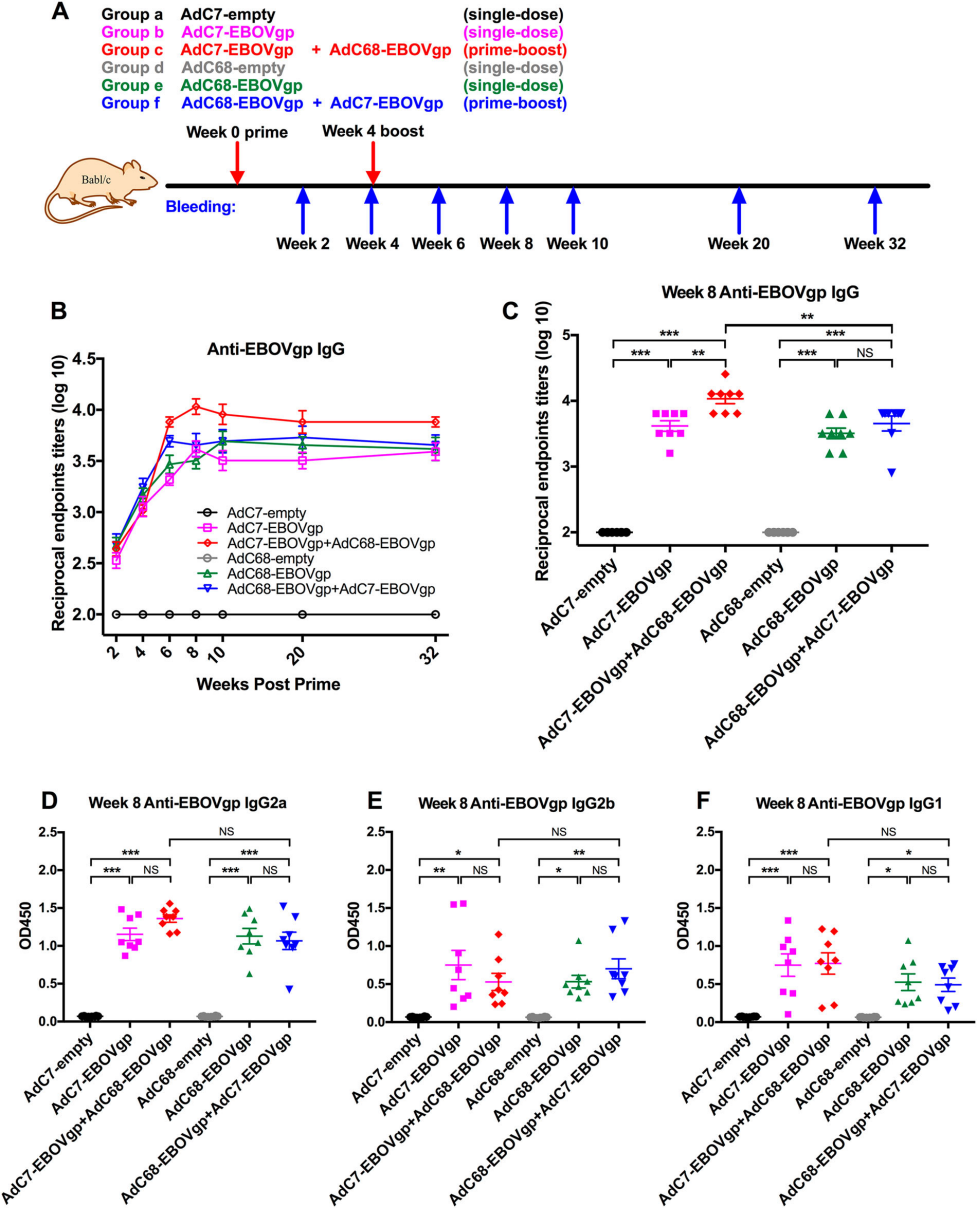
EBOV GP-specific IgG immune responses in immunized mice. (a) Chimpanzee adenoviral vaccine immunization and bleeding strategies in BALB/c mice. Six groups (*n* = 8) of BALB/c mice were i.m. injected with 2 × 10^10^ vp viruses. Blood samples were collected at different time points. (B) Kinetics of EBOV GP-specific total IgG reciprocal endpoints titres (log10) in 32 weeks post vaccination. Total IgG immune responses were measured by ELISA. (C) Anti-EBOV total IgG reciprocal endpoint titer (log10) of each immunized group at week 8 post vaccination. (D–F) represented EBOV GP-specific isotypes IgGs responses, IgG2a shown in (D), IgG2b shown in (E) and IgG1 shown in (F), all data were collected at week 8 post vaccination. The absorption values were detected at 450 nm. All data were displayed as mean ± SEM (standard error of mean). Significant differences were displayed as: NS, no significant differences; *P < 0.05; **P < 0.01; ***P < 0.001.

Female BALB/c mice aged approximately 6–8 weeks were obtained from Shanghai laboratory animal center and were housed in specific pathogen-free facilities at Institut Pasteur of Shanghai, Chinese Academy of Sciences, Shanghai, China. Mice were randomly divided into six groups (*n* = 8 or *n* = 5) and were immunized via i.m. injection with 2 × 10^10^ vp of different adenoviruses (Figure 1(A)) and prime-boost groups were boosted with the same dosage at week 4. All mice blood samples were collected at different time points to detect antibody titre and T cell responses (Figure 1(A)).

Five Rhesus monkeys (2 males and 3 females) weighing 3–6 kg and aged 3–5 years old were purchased from Suzhou XiShan Zhongke Laboratory Animal Company, China and raised at the clean facility in Non-Human Primates Experiment Platform of Institute of Neuroscience, Chinese Academy of Sciences. Vaccine group (*n* = 3, one male and two females) monkeys were immunized with 5 × 10^10^ vp of AdC7-EBOVgp at week 0 and 5 × 10^10^ vp of AdC68-EBOVgp at week 4 via the i.m. injection, while control group (*n* = 2, one male and one female) animals were vaccinated with the same dose of AdC7-empty at week 0 and AdC68-empty at week 4 (Figure 5(A)). Blood samples of monkeys were collected at week 0, 2, 4, 6, 8, 12, and 24.

### Anti-EBOV GP binding antibody detection

EBOV GP1 proteins were generated as previously described [25]. GP1 proteins (300 ng) were coated onto the bottom of each well of a 96-well ELISA plate (Costar) and incubated at 4°C for overnight. After blocking with 5% skim milk at 37°C for 2 h, the plates were washed three times with PBST and then added with serially diluted sera collected at different time points, at a starting dilution of 1:100 and were incubated at 37°C for another 2 h. Plates were washed five times with PBST and then incubated with 1:8000 dilutions of horseradish peroxidase(HRP)-conjugated anti-mouse IgG (A0168, Sigma–Aldrich), or 1:5000 dilutions of HRP-conjugated anti-monkey IgG (sc-2458, Santa Cruz Biotechnology), or 1: 5000 dilutions of HRP-conjugated anti-mouse IgG2a, IgG2b, and IgG1 (Southern Biotechnology, Birmingham, AL, USA) at 37°C for 1 h. This was followed by the washing steps and then the addition of the 3,3′,5,5′-Tetramethylbenzidine (TMB) substrate (New Cell & Molecular Biotech Co., Ltd, Suzhou, China) to observe the colour reaction, which was stopped using a 2 M sulphuric acid (H_2_SO_4_) solution. The absorbance at 450 nm was measured using a Varioskan Flash multimode reader (Thermo Scientific). The antibody endpoint titre was determined as the reciprocal of the highest sera dilution with an absorbance reading greater or equal to 0.1 OD unit above the absorbance of the pre-immune samples.

### EBOV GP neutralizing assays

Anti-EBOVgp neutralization antibody detection was performed by applying a pseudotyped lentivirus with EBOV GP membrane proteins, a firefly luciferase reporter gene and backbone of human immunodeficiency virus (HIV) as previously described [25,26]. Neutralizing assays were carried out as follows. Madin-Darby Canine Kidney (MDCK) cells (approximately 20,000 cells) were seeded into a 96-well flat-bottomed cell culture plate and cultured at 37°C in a humidified 5% CO_2_ incubator overnight. 50 µl 100 × TCID50 (50% tissue culture infective doses) of the EBOV pseudovirus were co-cultured with an equal volume of serially diluted heat-inactivated plasma samples at a starting dilution of 1:25 (murine plasma) or 1:20 (monkey plasma). After incubation at room temperature (RT) for 1 h, the mixtures were added back to the MDCK cells. Relative luciferase activity (RLA) was measured using the luciferase assay system (Promega) 48 h after infection. The formula for calculating the neutralizing activity is as follows: Neutralizing activity = (RLA of EBOV pseudovirus-infected cells–RLA of infected cells with plasma treated-EBOV pseudovirus)**/**(RLA of EBOV pseudovirus-infected cells) **× **100%. Neutralizing titer 50 (NT50) was defined as the highest reciprocal dilution of plasma samples with a neutralizing activity greater or equal to 50%.

### Chimpanzee adenovirus neutralization assays

Chimpanzee adenovirus-specific neutralizing antibodies were measured as described previously [27].

### ELISPOT assays

Murine peripheral blood mononuclear cells (PBMCs) were isolated as previously described [28]. Splenocytes were isolated from murine spleen by using 100 μm cell strainers, followed by treatment with Ammonium-Chloride-Potassium (ACK) lysis buffer (Beyotime) to remove the red blood cells. The isolated cells were then suspended in complete RPMI-1640 medium (Gibco) and counted for the following experiments. IFN-γ ELISPOT for mice was measured as previously described [28]. Briefly, 96-well PVDF plates (Millipore, Bedford, MA, USA) were pre-coated with 15 μg/ml anti-mouse IFN-γ coating antibody (An-18, Mabtech, Nacka, Sweden) at 4°C overnight, and then blocked with complete RPMI-1640 medium for 2 h at 37°C. Freshly isolated PBMCs or spleenocytes were transferred to the plates and stimulated with a peptides pool (0.5 μg/ml of each peptide) spanning the entire EBOV GP (GL Biochem Ltd, Shanghai, China), the peptides were 15-mers overlapping by 10 amino acids, and incubated for 48 h at 37°C and 5% CO_2_. Control stimuli were PBS (as negative control) or 10 μg/ml concanavalin A (as positive control). Subsequently, the plates were incubated with 1 μg/ml anti-mouse IFN-γ detection antibody (R4-6A2-biotin, Mabtech) at room temperature for 2 h and then with streptavidin-HRP (dilution 1:500, Mabtech) for 1 h. After washing, 100 μL/well of TMB substrate solution (Mabtech) were added and developed for 15–30 min until distinct spots emerge. The cytokine-secreting cell spots were imaged and counted on a CTL Immunospot reader (Cellular Technology Ltd, Shaker Heights, USA).

### EBOVLP challenge in immunized mice

An EBOVLP based reporter system containing EBOV VP40, NP, GP, and firefly luciferase proteins were prepared as previously described [19]. Briefly, pcDNA3.1-VP40, pcDNA3.1-NP, pcDNA3.1-GP, and pLenti6-Fluc plasmids were co-transfected into 293T cells with polyethylenimine transfection reagents. Supernatants containing EBOVLP were harvested after 48 h and ultracentrifuged at 27,000 rpm for 3 h through a 25% sucrose cushion. The crude purification of EBOVLP precipitate was resuspended in PBS and was ready for use. Six groups (*n* = 5) of BALB/c mice were immunized as described above. Immunized mice were challenged with EBOVLP containing 2 μg of VP40 through intravenous injection after 12 weeks. 12 h post-infection, mice were anesthetized using pentobarbital sodium anesthesia and injected with 1.5 mg/mouse of VivoGlo Luciferin (Promega) reconstituted in sterile PBS via the intraperitoneal route. *In vivo* imaging and bioluminescence measurement were performed using the IVIS SpectrumCT System (PerkinElmer). Average radiance (photons/s/cm2/sr) represent total flux (photons/sec or p/s) from each pixel inside the ROI (region of interest) / number of pixels or super pixels.

### Passive transfer of immune serum

Plasma of rhesus monkeys was collected at 2 weeks post boost (6 weeks after prime) as described above. Two groups (*n* = 5) of naïve BALB/c mice were intraperitoneally injected with 500 μL/mouse pooled sera of immunized group (AdC7-EBOVgp prime-AdC68-EBOVgp boost) or control group (AdC7-empty prime-AdC68-empty boost). 12 h post treatment, mice were challenged with EBOVLP and *in vivo* imaging and bioluminescence measurement were performed as described above.

### Statistical analysis

Statistical significance between groups was analysed using one-way analysis of variance (ANOVA) with LSD multiple comparison tests or Student’s t-test. The correlation between the immune protection and antibody responses was analysed using Pearson’s correlation analysis. *P* values < 0.05 were considered statistically significant. All statistical analyses were performed using the GraphPad Prism 7.0 (GraphPad Software, La Jolla, CA) software.

## Results

### Construction and expression of AdC7-EBOVgp and AdC68-EBOVgp

The full-length GP of Makona-C07 strain identified in the 2014 EBOV outbreak was chosen as antigen. The codon-optimized GP sequence under the regulation of cytomegalovirus (CMV) promoter was cloned into the E1-deleted region (ΔE1) of adenoviral vectors, AdC7 and AdC68, respectively (Supplementary Figure 1A). The recombinant viruses were rescued and propagated in HEK293 cells and viral particles (vp) were determined by the absorbance at 260 nm. AdC7-empty and AdC68-empty viruses with no insertion in ΔE1 were generated as negative controls using the same amplification method.

The EBOV GP expression levels of two vectors were assessed *in vitro* by Western blot. GP of both vectors was highly expressed in 10^10^ vp infected HEK293 cells, whereas no GP-specific band was detected in control vectors (Supplementary Figure 1B). These results demonstrated the successful construction of two recombinant adenoviruses expressing EBOV GP.

### EBOV GP-specific antibody responses in mice

Six groups of BALB/c mice were immunized intramuscularly (i.m.) with 2 × 10^10^ vp viruses, and Group c and Group f were boosted at week 4 with the same dosage of viruses (Figure 1(A)). Blood samples were collected at specific time points and sera were isolated to measured EBOV GP-specific antibodies. As depicted in Figure 1(B), immunization with either AdC7-EBOVgp (Group b or c) or AdC68-EBOVgp (Group e or f) induced GP-specific antibody responses at week 2 and stably increased at week 4. After the boost, the antibody titres in both AdC7/AdC68 (Group c) and AdC68/AdC7 (Group f) groups increased rapidly at week 6 when compared with a slight growth in the single shot groups (Group b and e) (Figure 1(B)). Regarding AdC7/AdC68 vaccination, the average antibody titre was peaked to the highest level of 12000 at week 8 (Figure 1(B,C)) and with some slight changes over time to maintain the high level of about 8000 at week 32 (Figure 1(B)). Although the antibody titres in AdC68/AdC7 group were higher after the boost, the mean titres remained approximately 5500, which were much lower than AdC7/AdC68 vaccination (Figure 1(B,C)). Comparison of the endpoint titres of all groups at week 8 is specified in Figure 1(C). These results indicated that both AdC7-EBOVgp and AdC68-EBOVgp could trigger EBOV GP specific immune responses in mice. AdC7/AdC68 prime-boost elicited much stronger and more durable antibody response than single-dose immunization and the AdC68/AdC7 prime-boost regimen.

IgG subtype in each group was also measured. As shown in Figure 1(D–F), stronger IgG2a, IgG2b, and IgG1 responses are observed at week 8 in four vaccine groups (Group b, c, e, and f), while nothing was detected in control groups (Group a and d). IgG isotype profile was further tested at week 20. IgG2a and IgG1 of four vaccine groups maintained the higher level and AdC7/AdC68 group was still above others (Supplementary Figure 2A, C and D). However, the levels of IgG2b were reduced in four vaccine groups than those at week 8 (Supplementary Figure 2B and D). Generally, IgG2a and IgG2b isotypes relate to Th1 dominant immune response and IgG1 relates to Th2 response activation in mice [29]. Hence, the results indicate that one-shot of AdC7 or AdC68 could induce both Th1 and Th2 immune responses.

**Figure 2.**
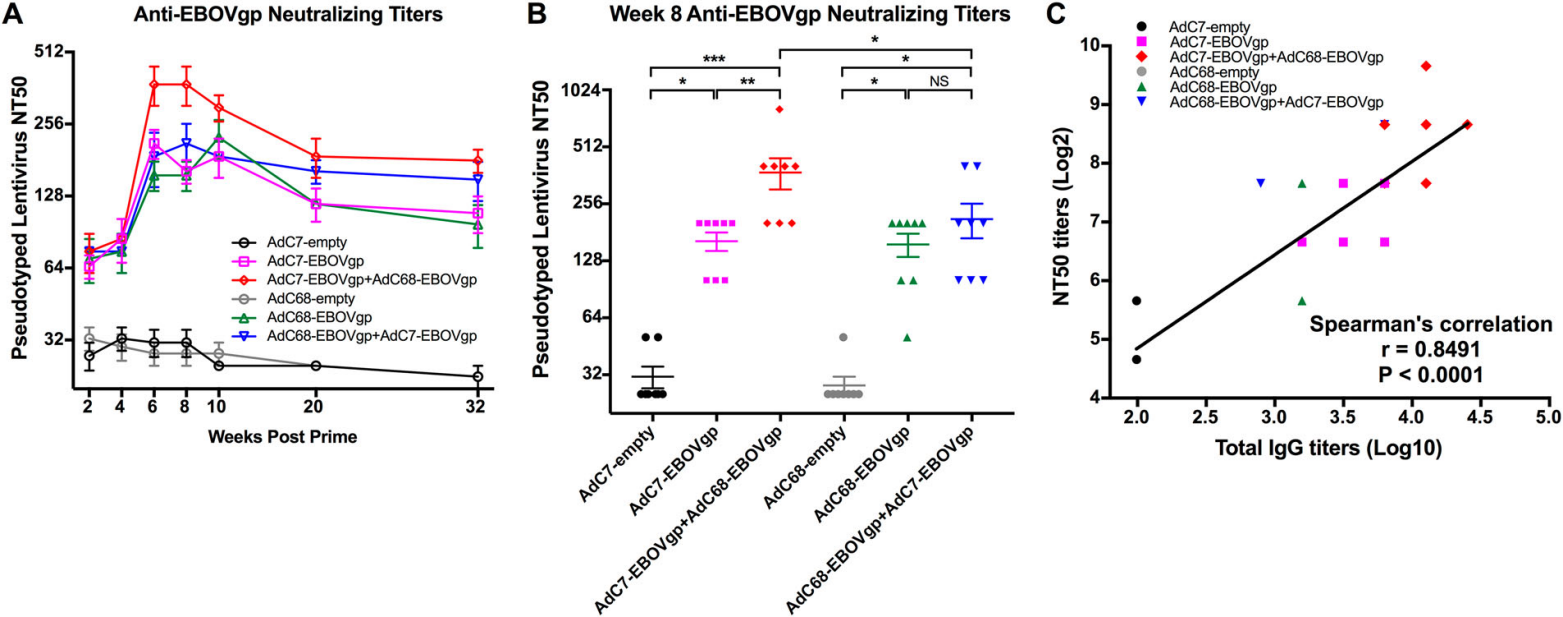
Neutralizing antibody responses in immunized mice. (A) Kinetics of EBOV GP specific neutralizing antibody responses NT50 in 32 weeks post vaccination. (B) NT50 responses of each immunized groups at week 8 post vaccination. (C) Correlation between NT50 titres and total IgG titres. Pearson’s correlation was applied in this analysis. All data were displayed as mean ± SEM. Significant differences were displayed as: NS, no significant differences; *P < 0.05; **P < 0.01; ***P < 0.001.

An EBOV pseudotype-based neutralization antibody titration assay (NTA) was developed in our lab and was used to determine the GP-specific NTA [25]. Consistent with the IgG antibody responses, four vaccine groups elicited higher NTA titres than the control groups after the first immunization (Figure 2(A)). After boost, the AdC7/AdC68 group triggered mean NTA titre to the highest level of 375 at week 6 (Figure 2(A,B)); the NTA titre decreased over time and was maintained at around 180 at week 32. Though the NTA titres in AdC68/AdC7 group were increased post-boost, they were still lower than those in the AdC7/AdC68 group (Figure 2(A,B)). Importantly, the NTA titres significantly correlated with the total IgG titres (*r* = 0.8491, *P* < 0.0001) (Figure 2(C)) and IgG2a (*r* = 0.8092, *P* < 0.0001) (Supplementary Figure 3A and B). Altogether, these results demonstrate that our AdC7 and AdC68 EBOV vaccine showed strong immunogenicity in the murine model.

### EBOV GP-specific T cell responses in mice

**Figure 3.**
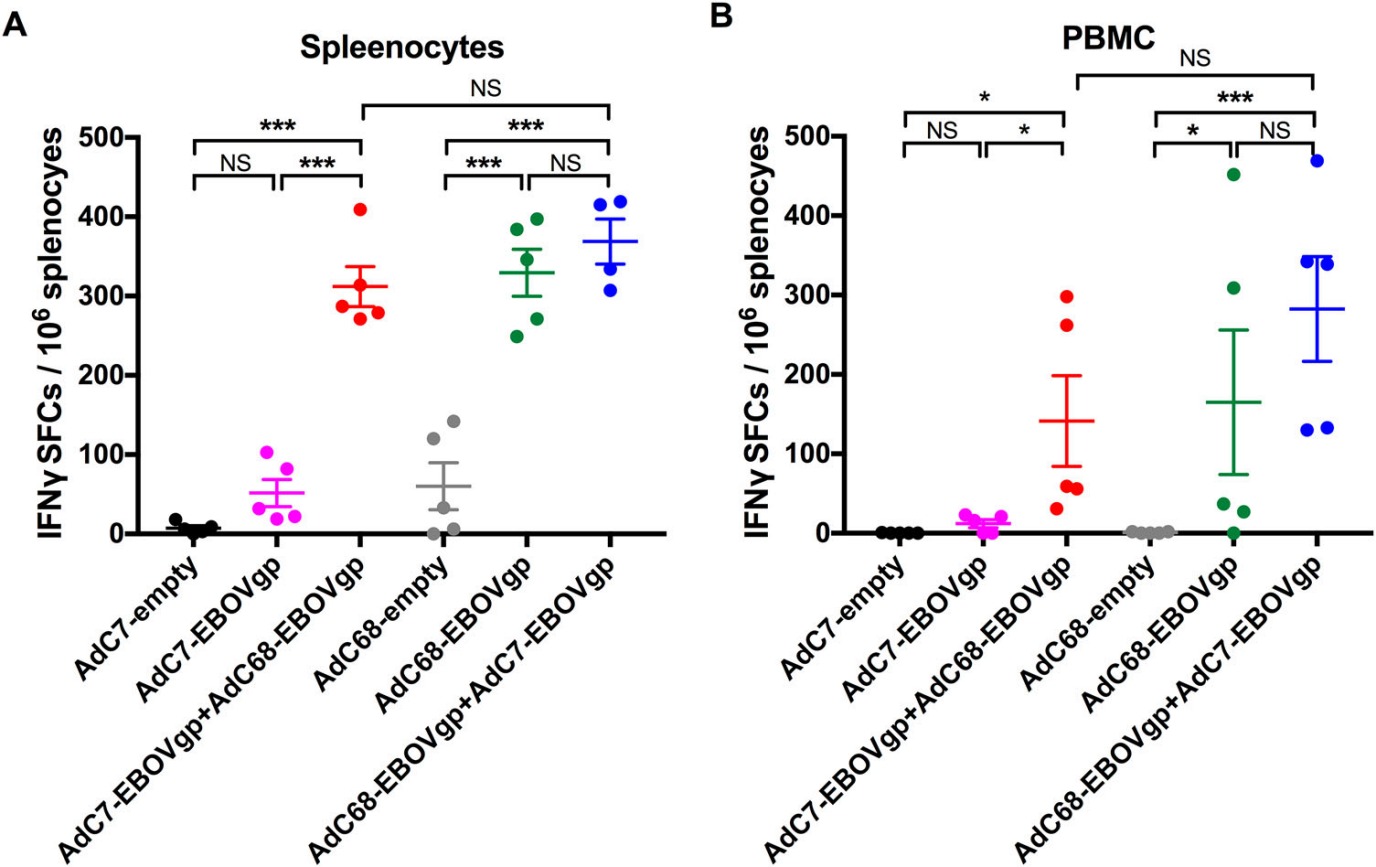
EBOV GP-specific T cell immune responses in immunized mice. Six groups (*n* = 5) of BALB/c mice were immunized with 2 × 10^10^ vp viruses via the i.m. injection. ELISPOT was performed to evaluate the ability of (A) PBMC and (B) splenocytes to secrete IFN-γ following stimulation with EBOV GP peptides pool at week 8 post immunization. All data were displayed as means ± SEM. Significant differences were displayed as: NS, no significant differences; *P < 0.05; **P < 0.01; ***P < 0.001.

Enzyme-linked immunospot (ELISPOT) assays were performed to measure murine T cell responses. As depicted in Figure 3(A,B), a set of mice was sacrificed at 8 weeks after vaccination and both PBMCs (peripheral blood mononuclear cells) and mouse splenocytes were harvested. Interferon gamma (IFN-γ) spot-forming cells (SFCs) in the AdC7/AdC68 group were much higher than those in the AdC7-EBOV group or the AdC7-empty group in both PBMCs and splenocytes. Surprisingly, the SFCs for IFN-γ in both AdC7/AdC68 group and AdC68/AdC7 group were at a similar level, whereas those in both were significantly higher than that in the AdC68-empty group.

### Protection against EBOVLP challenge in mice

**Figure 4.**
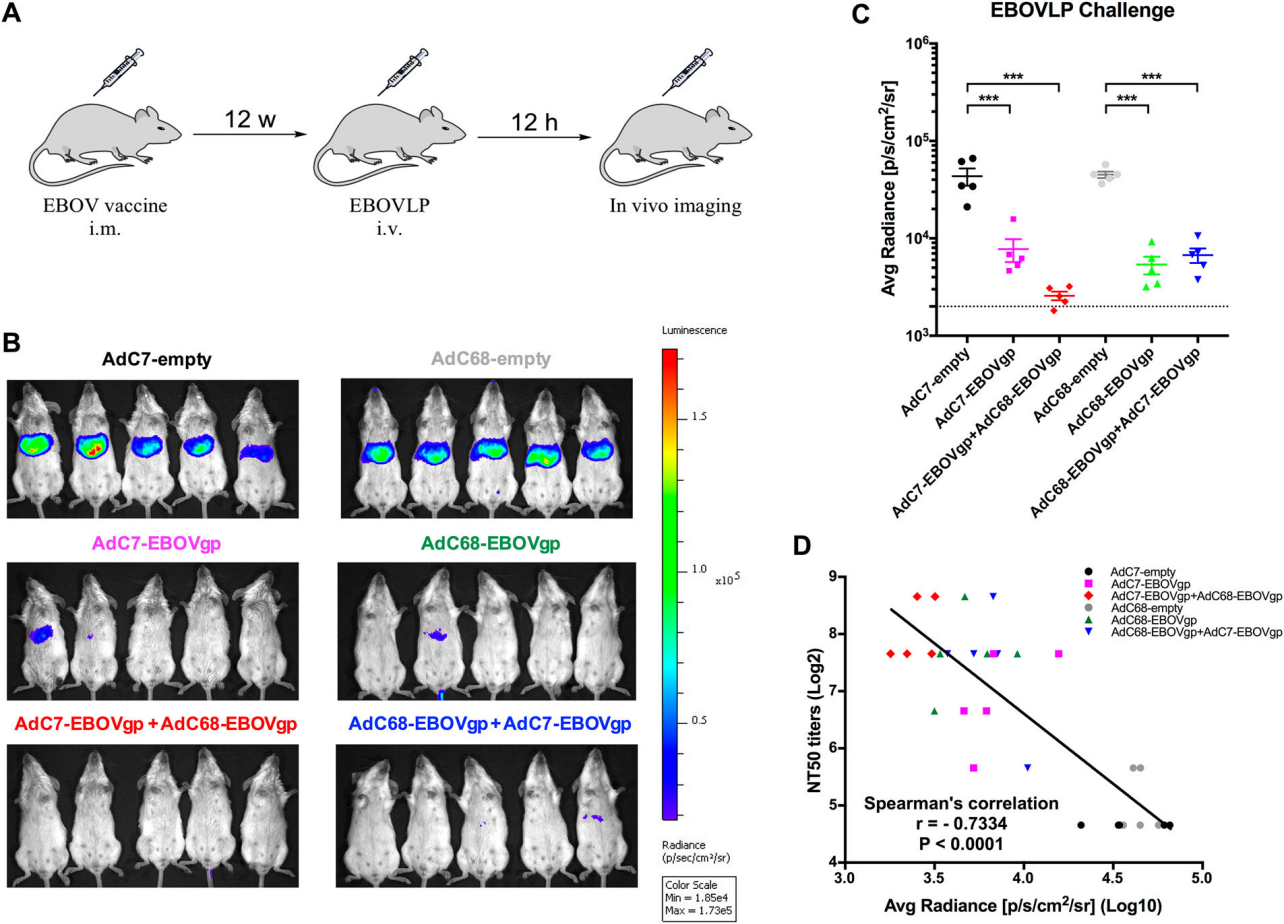
Protection against EBOVLP challenge in vaccinated mice. (A) Schematic diagram of EBOVLP challenge in mice. Six groups of BALB/c mice were immunized as described previously. After 12 weeks, EBOVLP was injected via i.v. route and *in vivo* imaging was performed 12 h post challenge. (B) *In vivo* bioluminescence signal images and (C) the measurement value of different murine groups were shown separately. The dotted line indicates the baseline, which is the bio-signal value of a non-immunized mouse. (D) Correlation between NT50 titres and bioluminescence signals of immunized mice. All data were displayed as mean ± SEM. Significant differences were displayed as: NS, no significant differences; *P < 0.05; **P < 0.01; ***P < 0.001.

Instead of the EBOV challenge that should be operated in biosafety level 4 (BSL-4) facility, the EBOVLP challenge performed in this study used EBOV-like particles expressing firefly-luciferase (Fluc) to validate the potential protection of the EBOV vaccine regimens in animals [19]. As shown in Figure 4(A), groups of BALB/c mice were immunized as described above and received EBOVLP via intravenous (i.v.) route 12 weeks post immunization. *In vivo* imaging and bioluminescence signal detection was performed after 12 h. The bio-signals in four vaccine groups were sharply declined compared with the control groups (Figure 4(B,C)). Particularly in the AdC7/AdC68 group, nearly no visible bio-signals were observed (Figure 4(B,C)). In addition, there was a significantly negative correlation between the NTA titres and bio-signals in mice (*r* = −0.7334, *P* < 0.0001) (Figure 4(D)). These results implied that the AdC7/AdC68 prime-boost exceeded AdC7 or AdC68 single vaccination or AdC68/AdC7 prime-boost in blocking EBOVLP infection in mice, and the protection is significantly correlated with neutralizing antibodies induced by vaccine regimen.

### EBOV GP-specific immune response in prime-boost rhesus macaques

**Figure 5.**
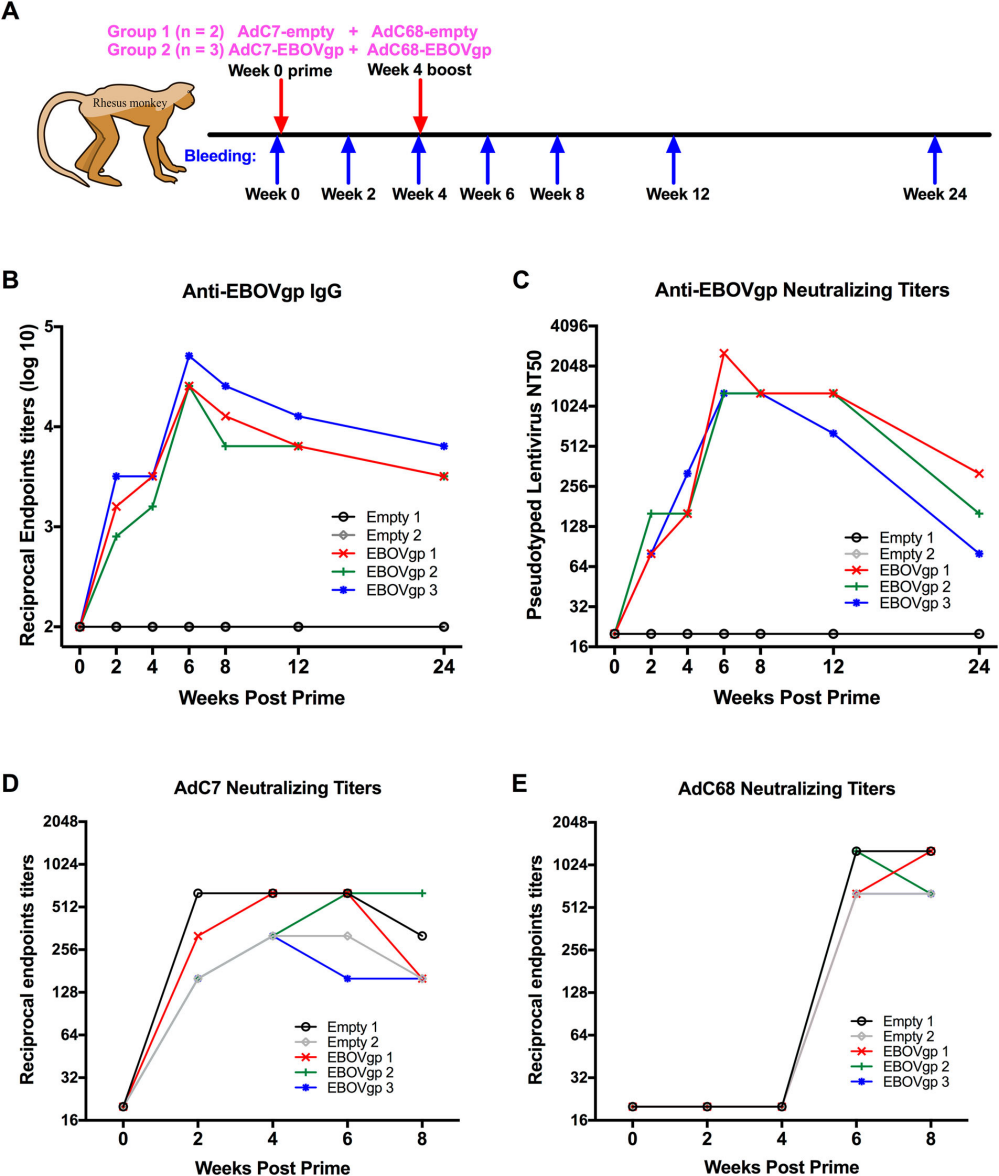
EBOV GP-specific antibody responses and chimpanzee Ad neutralizing antibody in rhesus macaques. (A) Five rhesus macaques were divided into two groups. Monkeys were primed with 5 × 10^10^ vp of AdC7-empty or AdC7-EBOVgp at week 0 and boosted with the same dose of AdC68-empty or AdC68-EBOVgp at week 4. Blood samples were collected at different time points. (B) Kinetics of EBOV GP specific total IgG reciprocal endpoints titers (log10) and (C) neutralizing antibody NT50 of monkeys at 24 weeks post-immunization were presented. Kinetics of (D) AdC7 and (E) AdC68 specific neutralizing activities of individual monkeys at 8 weeks post-immunization were presented. Empty 1 and Empty 2 represented two individual monkeys in the control group, while EBOVgp1, EBOVgp2, and EBOVgp3 represented three individual monkeys in the vaccine group.

The immunogenicity of AdC7/AdC68 prime-boost was particularly evaluated in non-human primates (NHPs). Five rhesus macaques were divided into two groups. As shown in Figure 5(B), total IgG titres of three individual monkeys in vaccine group were elevated to a high level after prime and intensely increased up to a peak value post boost, while the two control monkeys remained at a low level during the time. At week 6, IgG titres of three monkeys in vaccine group were 25600, 25600, and 51200, respectively, which were extremely higher compared with two controls, and they decreased over time to 3200, 3200, 6400 at week 24. Furthermore, the neutralizing antibody responses were measured in rhesus macaques. As shown in Figure 5(C), NTAs of three monkeys in vaccine group are raised after prime and intensively produced post-boost compared with two controls. The NTAs of those three were peaked to 1280, 2560, and 2560 at week 6 and maintained stability in week 12 and dropped to 320, 160 and 80 at week 24.

Adenovirus-specific neutralizing antibodies were assayed as well. All five rhesus macaques were screened with no neutralizing activity against both Ad vectors initially, suggesting that the monkeys had no AdC7 or AdC68 adenovirus infection before. AdC7 specific neutralizing titres of five monkeys were detected after prime and remained at a high level with little changes in 8 weeks (Figure 5(D)). The AdC68-specific neutralizing titres of five monkeys were induced only after the boost (Figure 5(E)). Numerically, the neutralizing titres of AdC68 (titres of the monkeys were 1280, 640, 1280, 640, 640, respectively) were higher than AdC7 (titres of the monkeys were 320, 160, 160, 640, 160, respectively) at week 8 (*p* < 0.05) (Figure 5(D,E)), which implies that the immunogenicity of AdC68 is superior to AdC7.

### Passive transfer of immune serum provided protection

**Figure 6.**
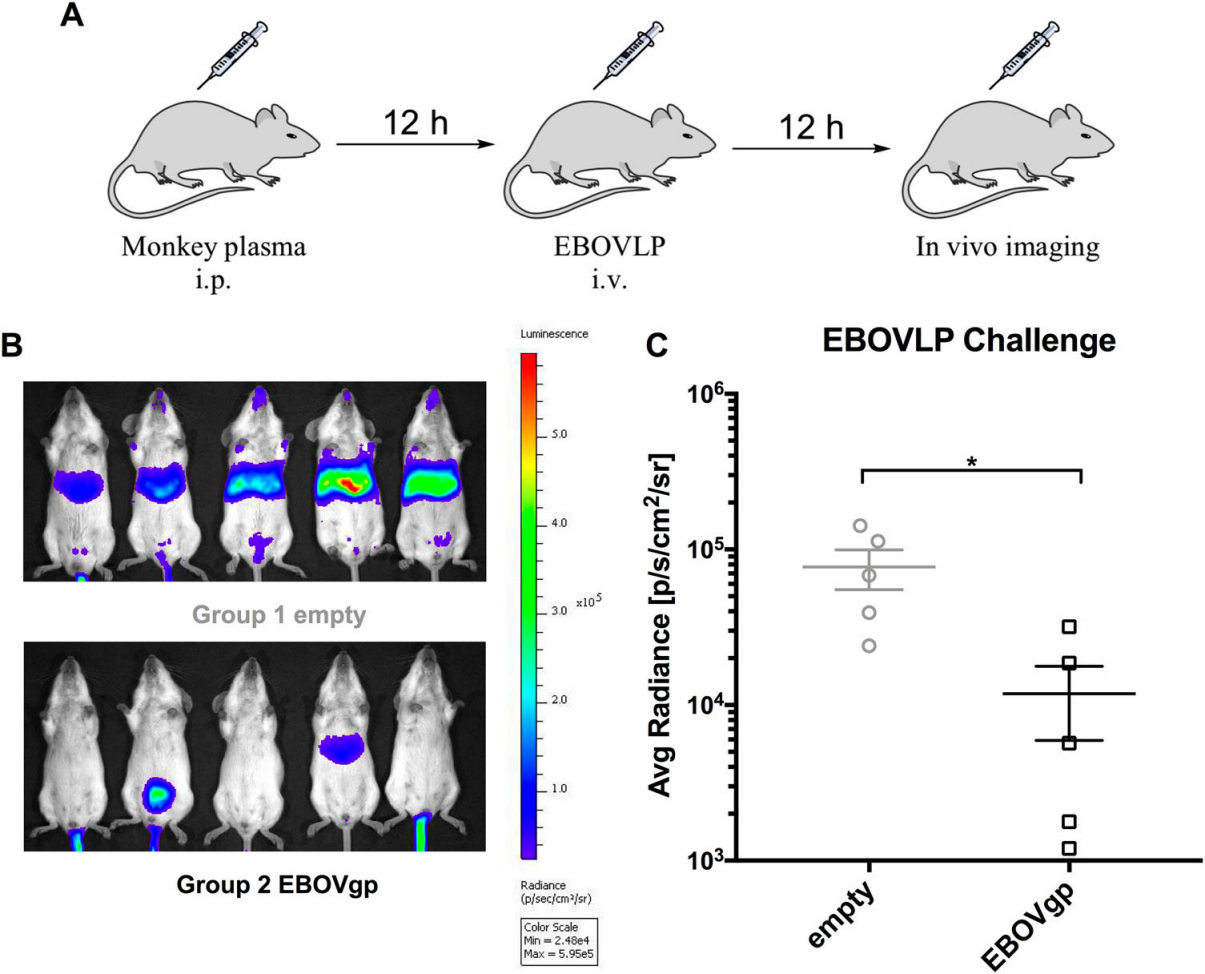
Antibodies from immunized rhesus macaques protected mice against EBOVLP. (A) Schematic diagram of EBOVLP challenge in mice. Two groups of mice received pooled plasma from vaccine and control monkeys via i.p. injection. After 12 h EBOVLP were injected into mice via i.v. route, and *in vivo* imaging was performed another 12 h post challenge. (B) *In vivo* bioluminescence signal images and (C) the measurement value of different murine groups were shown separately. All data were displayed as mean ± SEM. Significant differences were displayed as: NS, no significant differences; *P < 0.05; **P < 0.01; ***P < 0.001.

To investigate the potential protective effects of the antibodies induced by AdC7/AdC68 prime-boost regimen against EBOVLP challenge in monkey, sera from both vaccine and control monkeys were collected and passively transferred to naïve BALB/c mice. The treated mice were challenged with EBOVLP and performed *in vivo* bioluminescence imaging (Figure 6(A)). Mice received sera from the vaccinated monkeys displayed markedly weak bio-signals, while controls displayed strong bio-signals (Figure 6(B,C)). These results demonstrated that the antibodies induced by immunized macaques blocked EBOVLP infection at a certain extent in mice.

## Discussion

The EBOV envelope GP, which forms heterotrimeric spikes on the viral surface mediates macropinocytosis and plays a vital role in the virus-cell attachment and entry [30,31]. Therefore, GP has been chosen as a pivotal antigen for numerous EBOV vaccine studies. Many studies selected GP of EBOV Mayinga or Kikwit strain from previous outbreaks as antigens [32–34]. Despite the major homologous sequences among those strains, there are still many mutations in the strain derived from the 2014 outbreaks that differed from previous strains, which may cause different virulence [35–37]. Ever since the extensive impact of EVD outbreak in 2014, there are approximately 17,000 survivors suffering sequelae of symptoms post EBOV infection [5]. Moreover, the presence of EBOV RNA in semen of EVD survivors remains for several months, and the risk of re-emergence remains high [38]. Our vaccine that employed GP from Makona-C07 strain, might provide more powerful protection.

Adenoviral vectors, particularly the replication-deficient recombinant chimpanzee adenovirus, exhibit promising potential as great vaccine vectors against various diseases [39–42]. Generally, both AdC7 and AdC68 vectors stimulate highly intense transgene product-specific immunities in host, which are not to be dampened by pre-existing immunity to Ad5 [17,18,22,39]. These two vectors are closely related to human serotype 4 adenovirus, and they enter the cells through the coxsackie-adenovirus receptor and thus have similar tropism [17]. Regarding clinical development, these two vectors show great performance in terms of yields, genetic stabilities, and batch reproducibility. Predominantly, the E1-flanking sequences of AdC7 and AdC68 vectors differ from Ad5, preventing the outgrowth of replication-competent adenovirus in packing cell line, which has E1 regions of Ad5 for trans-complementation [18]. Therefore, replication-deficient AdC7 or AdC68 vectors are an ideal platform for vaccine development.

Nevertheless, only a few studies have compared these two vectors in terms of immunogenicity and immune efficacy in parallel when they serve as vaccine carriers. AdC68 is slightly superior to AdC7 in regarding to induce T cell responses when they were tested as SIV or malaria vaccine vectors [18,43], whereas AdC68 and AdC7 could elicit a similar level of malaria specific-antibody response, and conferred 92% and 83% protection against *Plasmodium berghei* challenge in mice 2 weeks post prime, respectively [43]. In this study, single immunization with AdC68 or AdC7 in mice stimulated comparable EBOV GP-specific antibody responses, while AdC68 induced higher T cell response compared with AdC7, which is consistent with the previous study [43]. When they vaccinated the rhesus monkeys, our results indicated that AdC68 triggered stronger neutralization antibody to AdC68 itself than that of AdC7. Taken together, the immunogenicity of AdC68 is likely to be better than AdC7.

We compared the AdC7/AdC68 to AdC68/AdC7 prime-boost, and attempted to determine which regimen might be better. As results showed in this study, AdC7/AdC68 prime-boost regimen surely induced stronger and more effective immune responses and protection efficacy than AdC68/AdC7 regimen. AdC7/AdC68 triggered long-term intense EBOV GP-specific IgG antibody, isotype IgGs (IgG2a, IgG2b, or IgG1), GP-specific neutralizing antibodies in both vaccinated mice and rhesus monkeys, which implies our chimpanzee adenoviral vectors-based prime-boost vaccine against EBOV may surpass the similar EBOV vaccines applied by a single immunization [10,11,44]. Besides the humoral response, T cell immune responses could also contribute to the protection against EBOV infection [45], and EBOV GP-specific CD8^+^ T cell responses were found in convalescent phase of EVD [46]. Our prime-boost vaccine could stimulate GP-specific T cell response at a certain degree in mice, which is consistent with other adenovirus-based EBOV vaccines [11,44]. Although the T cell immune responses enhanced the broad spectrum of immune protection, many studies have shown that the antibody response is sufficient for immune protection against Ebola virus infection [47–49].

BSL-4 facility is required for EBOV infection experiments, but it only exists in very few countries. Recently, an EBOV-like particles (EBOVLP) system was established via co-expression of GP, matrix protein VP40, nucleoprotein NP, and firefly luciferase in mammalian cells to mimic the virus challenge in BSL-4 free conditions [19]. This allows us to test the *in vivo* efficacy of EBOV vaccine rapidly even in BSL-2 laboratories. Furthermore, cynomolgus macaques and rhesus macaques are considered the standard animal model for Ebola and Marburg virus infection [50]. In this study, AdC7 prime-AdC68 boost could block EBOVLP entry in mice, and the prime-boosted rhesus macaques induced antibodies, which provided passive protection against EBOVLP. Therefore, it was found that our prime-boost vaccine could provide great potential protection against EBOV infection. Undoubtedly, EBOV isolate challenge in BSL-4 is still in demand for further verification.

In summary, we first exploited two heterologous chimpanzee Ad vectors as prime-boost vaccine against EBOV and discovered that AdC7/AdC68 prime-boost strategy surpassed the AdC68/AdC7 prime-boost or two single vaccinations. These findings indicate that our prime-boost vaccine might serve as a promising vaccine candidate against EBOV and warrant extensive testing for clinical usage.

## Supplementary Material

Supplemental Material

## References

[1] Bowen ET, Lloyd G, Harris WJ, et al. Viral haemorrhagic fever in southern Sudan and northern Zaire. Preliminary studies on the aetiological agent. Lancet. 1977 Mar 12;1(8011):571–573. PubMed PMID: 65662. doi: 10.1016/S0140-6736(77)92001-3 65662

[2] Messaoudi I, Amarasinghe GK, Basler CF. Filovirus pathogenesis and immune evasion: insights from Ebola virus and Marburg virus. Nat Rev Microbiol. 2015 Nov;13(11):663–676. DOI:10.1038/nrmicro3524 PubMed PMID: 26439085; PubMed Central PMCID: PMCPMC5201123. 26439085 PMC5201123

[3] Feldmann H, Jones S, Klenk HD, et al. Ebola virus: from discovery to vaccine. Nat Rev Immunol. 2003 Aug;3(8):677–685. DOI:10.1038/nri1154. PubMed PMID: 12974482. 12974482

[4] Baize S, Pannetier D, Oestereich L, et al. Emergence of Zaire Ebola virus disease in Guinea. N Engl J Med. 2014 Oct 9;371(15):1418–1425. DOI:10.1056/NEJMoa1404505. PubMed PMID: 24738640. 24738640

[5] WorldHealthOrganization. Ebola situation report 30 March 2016 [Report]. Available from: http://apps.who.int/ebola/current-situation/ebola-situation-report-30-march-2016

[6] Cenciarelli O, Gabbarini V, Pietropaoli S, et al. Viral bioterrorism: learning the lesson of Ebola virus in West Africa 2013–2015. Virus Res. 2015 Dec 2;210:318–326. DOI:10.1016/j.virusres.2015.09.002. PubMed PMID: 26359111. 26359111

[7] Martin JE, Sullivan NJ, Enama ME, et al. A DNA vaccine for Ebola virus is safe and immunogenic in a phase I clinical trial. Clin Vaccine Immunol. 2006 Nov;13(11):1267–1277. DOI:10.1128/CVI.00162-06. PubMed PMID: 16988008; PubMed Central PMCID: PMCPMC1656552. 16988008 PMC1656552

[8] Warfield KL, Swenson DL, Olinger GG, et al. Ebola virus-like particle-based vaccine protects nonhuman primates against lethal Ebola virus challenge. J Infect Dis. 2007 Nov 15;196(Suppl 2):S430–S437. DOI:10.1086/520583. PubMed PMID: 17940980. 17940980

[9] Regules JA, Beigel JH, Paolino KM, et al. A recombinant vesicular stomatitis virus Ebola vaccine. N Engl J Med. 2017 Jan 26;376(4):330–341. DOI:10.1056/NEJMoa1414216. PubMed PMID: 25830322; PubMed Central PMCID: PMCPMC5408576. 25830322 PMC5408576

[10] Zhu FC, Hou LH, Li JX, et al. Safety and immunogenicity of a novel recombinant adenovirus type-5 vector-based Ebola vaccine in healthy adults in China: preliminary report of a randomised, double-blind, placebo-controlled, phase 1 trial. Lancet. 2015 Jun 6;385(9984):2272–2279. DOI:10.1016/S0140-6736(15)60553-0. PubMed PMID: 25817373. 25817373

[11] Ledgerwood JE, DeZure AD, Stanley DA, et al. Chimpanzee adenovirus vector Ebola vaccine. N Engl J Med. 2017 Mar 9;376(10):928–938. DOI:10.1056/NEJMoa1410863. PubMed PMID: 25426834. 25426834

[12] Winslow RL, Milligan ID, Voysey M, et al. Immune responses to novel adenovirus type 26 and modified vaccinia virus Ankara-vectored Ebola vaccines at 1 Year. JAMA. 2017 Mar 14;317(10):1075–1077. DOI:10.1001/jama.2016.20644. PubMed PMID: 28291882. 28291882

[13] Ewer K, Rampling T, Venkatraman N, et al. A monovalent chimpanzee adenovirus Ebola vaccine boosted with MVA. N Engl J Med. 2016 Apr 28;374(17):1635–1646. DOI:10.1056/NEJMoa1411627. PubMed PMID: 25629663; PubMed Central PMCID: PMCPMC5798586. 25629663 PMC5798586

[14] Mercknewsroom. (2018). Merck begins rolling submission of licensure application for V920 (rVSVΔG-ZEBOV-GP) to U.S. Food and Drug Administration KENILWORTH, N.J. Available from: Available from: https://www.mrknewsroom.com/news-release/ebola/merck-begins-rolling-submission-licensure-application-v920-rvsvΔg-zebov-gp-us-foo

[15] CFDA. (2017). The first recombinant Ebola virus vaccine was approved for new drug registration. Available from: http://samr.cfda.gov.cn/WS01/CL0050/178705.html

[16] Shukarev G, Callendret B, Luhn K, et al. A two-dose heterologous prime-boost vaccine regimen eliciting sustained immune responses to Ebola Zaire could support a preventive strategy for future outbreaks. Hum Vaccin Immunother. 2017 Feb;13(2):266–270. DOI:10.1080/21645515.2017.1264755. PubMed PMID: 27925844; PubMed Central PMCID: PMCPMC5328205. 27925844 PMC5328205

[17] Farina SF, Gao GP, Xiang ZQ, et al. Replication-defective vector based on a chimpanzee adenovirus. J Virol. 2001 Dec;75(23):11603–11613. DOI:10.1128/JVI.75.23.11603-11613.2001. PubMed PMID: 11689642; PubMed Central PMCID: PMCPMC114747. 11689642 PMC114747

[18] Tatsis N, Tesema L, Robinson ER, et al. Chimpanzee-origin adenovirus vectors as vaccine carriers. Gene Ther. 2006 Mar;13(5):421–429. DOI:10.1038/sj.gt.3302675. PubMed PMID: 16319951. 16319951

[19] Li D, Chen T, Hu Y, et al. An Ebola virus-like particle-based reporter system Enables Evaluation of Antiviral Drugs In vivo under Non-biosafety level 4 conditions. J Virol. 2016 Oct 1;90(19):8720–8728. DOI:10.1128/JVI.01239-16. PubMed PMID: 27440895; PubMed Central PMCID: PMCPMC5021419. 27440895 PMC5021419

[20] Vitelli A, Folgori A, Scarselli E, et al. Chimpanzee adenoviral vectors as vaccines - challenges to move the technology into the fast lane. Expert Rev Vaccines. 2017 Dec;16(12):1241–1252. DOI:10.1080/14760584.2017.1394842. PubMed PMID: 29047309. 29047309

[21] Wang Y, Li J, Hu Y, et al. Ebola vaccines in clinical trial: the promising candidates. Hum Vaccin Immunother. 2017 Jan 2;13(1):153–168. DOI:10.1080/21645515.2016.1225637. PubMed PMID: 27764560; PubMed Central PMCID: PMCPMC5287303. 27764560 PMC5287303

[22] Kobinger GP, Feldmann H, Zhi Y, et al. Chimpanzee adenovirus vaccine protects against Zaire Ebola virus. Virology. 2006 Mar 15;346(2):394–401. DOI:10.1016/j.virol.2005.10.042. PubMed PMID: 16356525. 16356525

[23] Cheng T, Song Y, Zhang Y, et al. A novel oncolytic adenovirus based on simian adenovirus serotype 24. Oncotarget. 2017 Apr 18;8(16):26871–26885. DOI:10.18632/oncotarget.15845. PubMed PMID: 28460470; PubMed Central PMCID: PMCPMC5432303. 28460470 PMC5432303

[24] Yang Y, Chi Y, Tang X, et al. Rapid, efficient, and Modular Generation of adenoviral vectors via Isothermal Assembly. Curr Protoc Mol Biol. 2016 04 January;113(1):16.26.1–16.26.8. doi: 10.1002/0471142727.mb1626s113 31773916

[25] Chen T, Li D, Song Y, et al. A heterologous prime-boost Ebola virus vaccine regimen induces durable neutralizing antibody response and prevents Ebola virus-like particle entry in mice. Antiviral Res. 2017 Sep;145:54–59. DOI:10.1016/j.antiviral.2017.07.009. PubMed PMID: 28733113. 28733113

[26] Zhang Q, Gui M, Niu X, et al. Potent neutralizing monoclonal antibodies against Ebola virus infection. Sci Rep. 2016 May 16;6:25856. DOI:10.1038/srep25856. PubMed PMID: 27181584; PubMed Central PMCID: PMCPMC4867612. 27181584 PMC4867612

[27] Wang X, Xing M, Zhang C, et al. Neutralizing antibody responses to enterovirus and adenovirus in healthy adults in China. Emerg Microbes Infect. 2014 May;3(5):e30. DOI:10.1038/emi.2014.30. PubMed PMID: 26038738; PubMed Central PMCID: PMCPMC4051363. 26038738 PMC4051363

[28] Wang X, Fu W, Yuan S, et al. Both haemagglutinin-specific antibody and T cell responses induced by a chimpanzee adenoviral vaccine confer protection against influenza H7N9 viral challenge. Sci Rep. 2017 May 12;7(1):1854. DOI:10.1038/s41598-017-02019-1. PubMed PMID: 28500340; PubMed Central PMCID: PMCPMC5431854. 28500340 PMC5431854

[29] Stevens TL, Bossie A, Sanders VM, et al. Regulation of antibody isotype secretion by subsets of antigen-specific helper T cells. Nature. 1988 Jul 21;334(6179):255–258. DOI:10.1038/334255a0. PubMed PMID: 2456466. 2456466

[30] Takada A, Watanabe S, Ito H, et al. Downregulation of beta1 integrins by Ebola virus glycoprotein: implication for virus entry. Virology. 2000 Dec 5;278(1):20–26. DOI:10.1006/viro.2000.0601. PubMed PMID: 11112476. 11112476

[31] Rivera A, Messaoudi I. Molecular mechanisms of Ebola pathogenesis. J Leukoc Biol. 2016 Nov;100(5):889–904. DOI:10.1189/jlb.4RI0316-099RR. PubMed PMID: 27587404. 27587404 PMC6608070

[32] Geisbert TW, Daddario-Dicaprio KM, Lewis MG, et al. Vesicular stomatitis virus-based Ebola vaccine is well-tolerated and protects immunocompromised nonhuman primates. PLoS Pathog. 2008 Nov;4(11):e1000225. DOI:10.1371/journal.ppat.1000225. PubMed PMID: 19043556; PubMed Central PMCID: PMCPMC2582959. 19043556 PMC2582959

[33] Marzi A, Ebihara H, Callison J, et al. Vesicular stomatitis virus-based Ebola vaccines with improved cross-protective efficacy. J Infect Dis. 2011 Nov;204(Suppl 3):S1066–S1074. DOI:10.1093/infdis/jir348. PubMed PMID: 21987743; PubMed Central PMCID: PMCPMC3203393. 21987743 PMC3203393

[34] Marzi A, Robertson SJ, Haddock E, et al. EBOLA VACCINE. VSV-EBOV rapidly protects macaques against infection with the 2014/15 Ebola virus outbreak strain. Science. 2015 Aug 14;349(6249):739–742. DOI:10.1126/science.aab3920. PubMed PMID: 26249231. 26249231 PMC11040598

[35] Schuit M, Miller DM, Reddick-Elick MS, et al. Differences in the Comparative stability of Ebola virus Makona-C05 and Yambuku-Mayinga in blood. PLoS One. 2016;11(2):e0148476. DOI:10.1371/journal.pone.0148476. PubMed PMID: 26849135; PubMed Central PMCID: PMCPMC4744009. 26849135 PMC4744009

[36] Marzi A, Feldmann F, Hanley PW, et al. Delayed disease progression in cynomolgus macaques infected with Ebola virus Makona strain. Emerg Infect Dis. 2015 Oct;21(10):1777–1783. DOI:10.3201/eid2110.150259. PubMed PMID: 26402165; PubMed Central PMCID: PMCPMC4593438. 26402165 PMC4593438

[37] Wong G, Qiu X, de La Vega MA, et al. Pathogenicity comparison between the Kikwit and Makona Ebola virus variants in rhesus macaques. J Infect Dis. 2016 Oct 15;214(suppl 3):S281–S289. DOI:10.1093/infdis/jiw267. PubMed PMID: 27651412; PubMed Central PMCID: PMCPMC5050479. 27651412 PMC5050479

[38] Deen GF, Broutet N, Xu W, et al. Ebola RNA persistence in semen of Ebola virus disease survivors – final report. N Engl J Med. 2017 Oct 12;377(15):1428–1437. DOI:10.1056/NEJMoa1511410. PubMed PMID: 26465681; PubMed Central PMCID: PMCPMC5798881. 26465681 PMC5798881

[39] Xiang Z, Gao G, Reyes-Sandoval A, et al. Novel, chimpanzee serotype 68-based adenoviral vaccine carrier for induction of antibodies to a transgene product. J Virol. 2002 Mar;76(6):2667–2675. PubMed PMID: 11861833; PubMed Central PMCID: PMCPMC135983. doi: 10.1128/JVI.76.6.2667-2675.2002 11861833 PMC135983

[40] Tatsis N, Ertl HC. Adenoviruses as vaccine vectors. Mol Ther. 2004 Oct;10(4):616–629. DOI:10.1016/j.ymthe.2004.07.013. PubMed PMID: 15451446. 15451446 PMC7106330

[41] Ewer K, Sebastian S, Spencer AJ, et al. Chimpanzee adenoviral vectors as vaccines for outbreak pathogens. Hum Vaccin Immunother. 2017 Dec 2;13(12):3020–3032. DOI:10.1080/21645515.2017.1383575. PubMed PMID: 29083948; PubMed Central PMCID: PMCPMC5718829. 29083948 PMC5718829

[42] Stanley DA, Honko AN, Asiedu C, et al. Chimpanzee adenovirus vaccine generates acute and durable protective immunity against ebolavirus challenge. Nat Med. 2014 Oct;20(10):1126–1129. DOI:10.1038/nm.3702. PubMed PMID: 25194571. 25194571

[43] Reyes-Sandoval A, Sridhar S, Berthoud T, et al. Single-dose immunogenicity and protective efficacy of simian adenoviral vectors against *Plasmodium berghei*. Eur J Immunol. 2008 Mar;38(3):732–741. DOI:10.1002/eji.200737672. PubMed PMID: 18266272. 18266272

[44] Feng Y, Li C, Hu P, et al. An adenovirus serotype 2-vectored ebolavirus vaccine generates robust antibody and cell-mediated immune responses in mice and rhesus macaques. Emerg Microbes Infect. 2018 Jun 6;7(1):101. DOI:10.1038/s41426-018-0102-5. PubMed PMID: 29872043; PubMed Central PMCID: PMCPMC5988821. 29872043 PMC5988821

[45] Sullivan NJ, Hensley L, Asiedu C, et al. CD8+ cellular immunity mediates rAd5 vaccine protection against Ebola virus infection of nonhuman primates. Nat Med. 2011 Aug 21;17(9):1128–1131. DOI:10.1038/nm.2447. PubMed PMID: 21857654. 21857654

[46] Dahlke C, Lunemann S, Kasonta R, et al. Comprehensive characterization of cellular immune responses following Ebola virus infection. J Infect Dis. 2017 Jan 15;215(2):287–292. DOI:10.1093/infdis/jiw508. PubMed PMID: 27799354. 27799354

[47] Wong G, Richardson JS, Pillet S, et al. Immune parameters correlate with protection against ebola virus infection in rodents and nonhuman primates. Sci Transl Med. 2012 Oct 31;4(158):158ra146. DOI:10.1126/scitranslmed.3004582. PubMed PMID: 23115355; PubMed Central PMCID: PMCPMC3789651. PMC3789651 23115355

[48] Qiu X, Audet J, Wong G, et al. Sustained protection against Ebola virus infection following treatment of infected nonhuman primates with ZMAb. Sci Rep. 2013 Nov 28;3:3365. DOI:10.1038/srep03365. PubMed PMID: 24284388; PubMed Central PMCID: PMCPMC3842534. 24284388 PMC3842534

[49] Qiu X, Wong G, Audet J, et al. Reversion of advanced Ebola virus disease in nonhuman primates with ZMapp. Nature. 2014 Oct 2;514(7520):47–53. DOI:10.1038/nature13777. PubMed PMID: 25171469; PubMed Central PMCID: PMCPMC4214273. 25171469 PMC4214273

[50] Geisbert TW, Strong JE, Feldmann H. Considerations in the use of nonhuman primate models of Ebola virus and Marburg virus infection. J Infect Dis. 2015 Oct 1;212(Suppl 2):S91–S97. DOI:10.1093/infdis/jiv284. PubMed PMID: 26063223; PubMed Central PMCID: PMCPMC4564553. 26063223 PMC4564553

