# Supplementary figures and images for: Chimpanzee adenoviral vector prime-boost regimen elicits potent immune responses against Ebola virus in mice and rhesus macaques
Source: Emerg Microbes Infect. 2019 Jul 24;8(1):1086–97. doi: 10.1080/22221751.2019.1644968 (PMC6711196; doi:10.1080/22221751.2019.1644968)

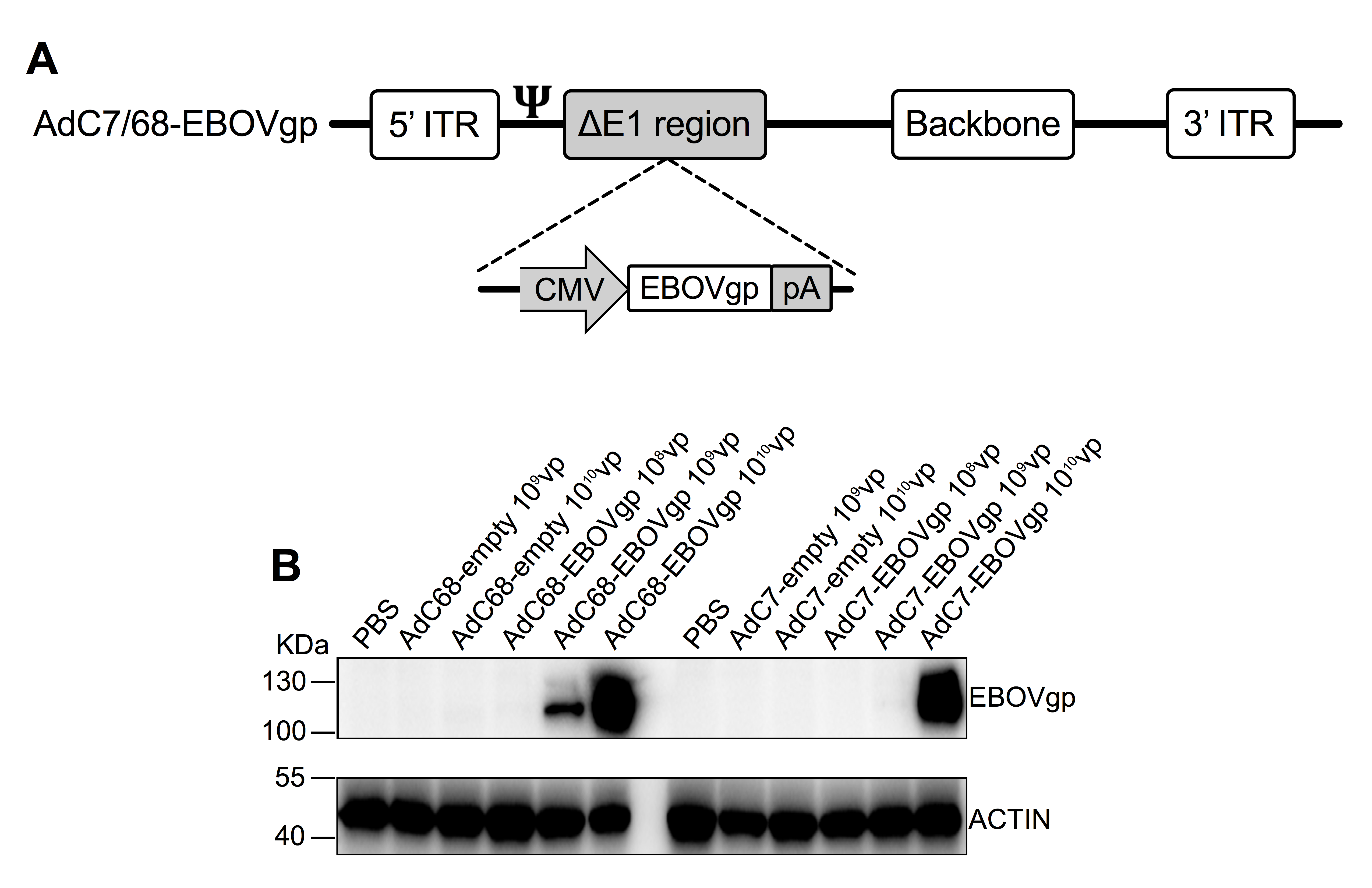

Supplement: Supplemental Material [file TEMI_A_1644968_SM6452.zip › Fig_S1.tiff]

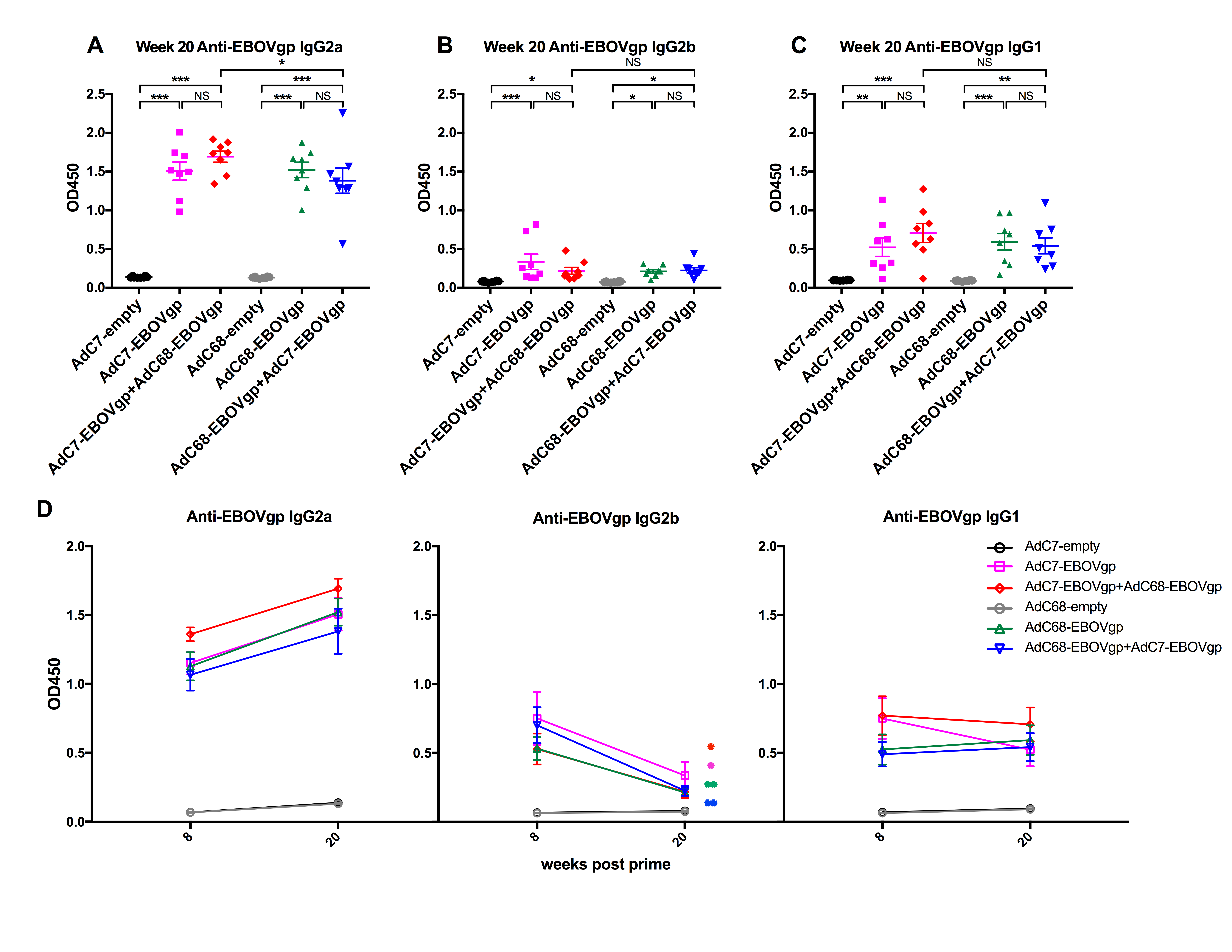

Supplement: Supplemental Material [file TEMI_A_1644968_SM6452.zip › Fig_S2.tiff]

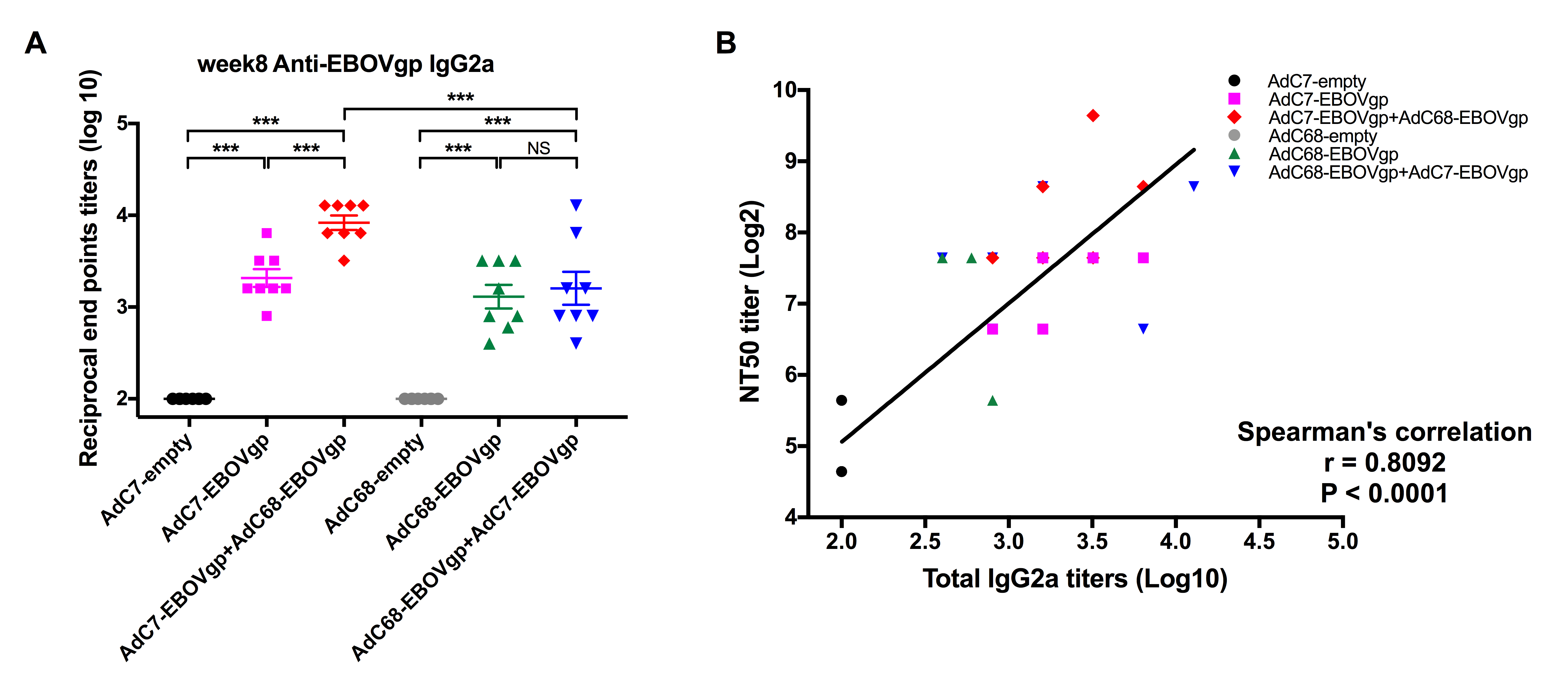

Supplement: Supplemental Material [file TEMI_A_1644968_SM6452.zip › Fig_S3.tiff]
